# Safety in Teletriage by Nurses and Physicians in the United States and Israel: Narrative Review and Qualitative Study

**DOI:** 10.2196/50676

**Published:** 2024-03-25

**Authors:** Motti Haimi, Sheila Quilter Wheeler

**Affiliations:** 1 Rappaport Faculty of Medicine Technion – Israel Institute of Technology Haifa Israel; 2 Health Systems Management Department The Max Stern Yezreel Valley College Emek Yezreel Israel; 3 Meuhedet Healthcare Services – North District Tel Aviv Israel; 4 TeleTriage Systems San Anselmo, CA United States

**Keywords:** telephone triage, teletriage, telehealth, telemedicine, safety, system error, human error, triage, outcome, patient safety

## Abstract

**Background:**

The safety of telemedicine in general and telephone triage (teletriage) safety in particular have been a focus of concern since the 1970s. Today, telehealth, now subsuming teletriage, has a basic structure and process intended to promote safety. However, inadequate telehealth systems may also compromise patient safety. The COVID-19 pandemic accelerated rapid but uneven telehealth growth, both technologically and professionally. Within 5-10 years, the field will likely be more technologically advanced; however, these advances may still outpace professional standards. The need for an evidence-based system is crucial and urgent.

**Objective:**

Our aim was to explore ways that developed teletriage systems produce safe outcomes by examining key system components and questioning long-held assumptions.

**Methods:**

We examined safety by performing a narrative review of the literature using key terms concerning patient safety in teletriage. In addition, we conducted system analysis of 2 typical formal systems, physician led and nurse led, in Israel and the United States, respectively, and evaluated those systems’ respective approaches to safety. Additionally, we conducted in-depth interviews with representative physicians and 1 nurse using a qualitative approach.

**Results:**

The review of literature indicated that research on various aspects of telehealth and teletriage safety is still sparse and of variable quality, producing conflicting and inconsistent results. Researchers, possibly unfamiliar with this complicated field, use an array of poorly defined terms and appear to design studies based on unfounded assumptions. The interviews with health care professionals demonstrated several challenges encountered during teletriage, mainly making diagnosis from a distance, treating unfamiliar patients, a stressful atmosphere, working alone, and technological difficulties. However, they reported using several measures that help them make accurate diagnoses and reasonable decisions, thus keeping patient safety, such as using their expertise and intuition, using structured protocols, and considering nonmedical factors and patient preferences (shared decision-making).

**Conclusions:**

Remote encounters about acute, worrisome symptoms are time sensitive, requiring decision-making under conditions of uncertainty and urgency. Patient safety and safe professional practice are extremely important in the field of teletriage, which has a high potential for error. This underregulated subspecialty lacks adequate development and substantive research on system safety. Research may commingle terminology and widely different, ill-defined groups of decision makers with wide variation in decision-making skills, clinical training, experience, and job qualifications, thereby confounding results. The rapid pace of telehealth’s technological growth creates urgency in identifying safe systems to guide developers and clinicians about needed improvements.

## Introduction

### Definitions and Terminology

*Telemedicine* refers to the electronic transmission of medical data from one source to another to promote clinical health. A wide range of services and applications, including 2-way video, email, smartphones, and other communications technology, are included in telemedicine. With the aid of these technologies, patients and caregivers who are geographically separated can communicate and receive treatment, consultation, follow-up, counseling, and health education, as well as engage in medical intervention, monitoring, and remote hospitalization [1,2]. The biggest benefits of telemedicine, aside from cost savings, are expanding patient access to treatment, expanding the availability of medical services, and improving clinicians’ efficacy [3].

The delivery and facilitation of health and health-related services, such as medical care, provider and patient education, health information services, and self-care, using telecommunications and digital communication technology is known as *telehealth*.

Although telehealth and telemedicine are frequently used synonymously, the term “telehealth” is used as an umbrella term to refer to all aspects and activities of health care and the health care system that are carried out via telecommunications technology, as opposed to the more specific term “telemedicine,” which only refers to the practice of medicine remotely [4,5].

*Triage* is the process of classifying and prioritizing symptoms. Based on quality, and in the context of health services, triage refers to the process of ranking patients according to their need for care. Using triage services can lower health care costs by preventing patients from making needless and expensive trips to emergency departments (EDs) and by assisting them with self-care and informal care while the doctor is away [6,7].

*Telephone triage* (assessment and triage of symptoms by telephone) predates telehealth by about 50 years. In the past 5-10 years, the broad industry of telehealth has subsumed telephone triage, which has quickly evolved into *teletriage* to include a wide range of high-tech features (video, biotelemetry, and patient wearables) to enhance remote, brief, but urgent encounters [6,8].

Teletriage is an unscheduled, brief (2-10 minutes), urgent encounter (by telephone only) initiated by patients seeking an estimate of symptom urgency and triage by a clinician to get an urgent on-site evaluation and definitive diagnosis [6,8].

*Televisits* (via video technology) are now a common substitute for a face-to-face medical appointment and may be 20-30 minutes in length.

Definitions and terms related to telehealth and teletriage are included in Multimedia Appendix 1.

### Teletriage: History and Characteristics

Wheeler et al [8] defined teletriage as the complex process of remotely assessing acute, worrisome symptoms to estimate their urgency and to render clinical advice and triage for further evaluation and diagnosis, as appropriate. The goal is to ensure the safe, timely, and appropriate disposition of patient symptoms remotely. This service is accomplished with remote encounters by telephone or real-time video (including biotelemetry). A disposition is a directive from clinician to patient about when and where to be further evaluated and treated. It may also include a risk estimation statement, such as “your symptoms sound urgent,” to both inform and motivate patients to comply with the medical advice [8].

Historically, the need for teletriage became an issue when health maintenance organizations (HMOs) realized that they could be more cost-effective by conserving on-site appointments for the sickest patients, which is a form of triage and a way to control access [9].

The overarching goal of medical care (systems and processes) is to use valid, reliable components and experienced trained clinicians to produce safe outcomes. Since the 1970s, clinicians have informally performed teletriage in ambulatory care settings ranging from urgent care and EDs, physician offices, clinics, and student health centers to disease management and ambulatory surgery. Beginning in the midnineties, teletriage and the telehealth industry began developing early systems [10].

One description of teletriage [10] is that it is a time-sensitive, complex, human-technology hybrid process of remote medical decision-making. Currently and in the future, a range of technologies will provide a range of information. On the continuum of care, teletriage can now be acknowledged as the entry point to clinical care. It legitimately qualifies as “prehospital care.”

By discussing treatment alternatives and the need for care, teletriage aims to identify the most appropriate degree of care that is needed. These alternatives could involve self-care or informal care, normal or emergency doctor visits, prompt referral to the ED or emergency clinics, or ambulance dispatch, depending on the data collected during evaluations. To support self-care and informal care, teletriage services may also entail providing information and help for difficult medical decisions, as well as managing symptoms. In a variety of medical facilities, nurses or doctors conduct teletriage [11,12].

The teletriage system, which is primarily run by nurses, determines the level of medical urgency and the type of health care that is needed when patients are contacted by telephone. This system is crucial for delivering affordable, effective, and secure health care [13].

The decision-making process is difficult and stressful in the emergency teletriage scenario because decisions must be made quickly and are dependent on nonvisible, unreliable, and incomplete information and nonvisible indicators. Additionally, patients’ capacities for clear symptom communication differ, particularly when young patients are involved. The lack of precise criteria for making the decision further increases the difficulty of the decision-making process [14,15].

Over the past few years, numerous Western nations and sizable corporations have started to offer primary health care services after regular business hours. In 2020, the COVID-19 pandemic accelerated telehealth growth exponentially. Almost overnight, telehealth rapidly became an established, essential service [16].

Currently, many US health plans provide advice lines. These services are advertised as a benefit of patients’ health plans. Advice lines offer clinicians’ advice for patients who have concerns about acute or worrisome symptoms who are calling from home. A telephone call to an after-hours advice line is typically the patients’ first attempt to gain access—a medical consultation for a symptom that patients interpret as urgent. However, standards are still lacking for clinical decision makers, their experience, qualifications, clinical training, and practice [8,17,18].

Once considered embryonic, telehealth now appears to be in an adolescent phase. It is rapidly and erratically growing, and technology is outpacing clinical standards. Telehealth appears to be undergoing an identity crisis [9].

Opposing forces—technology, cost containment, and safe clinical practice—now struggle to claim control of the field, one so new that regulation cannot keep pace. There are inherent risks in the clinical task—remote, rapid, clinical decision-making using software that serves technological interests but may not serve clinical safety [18].

In the United States, these forces are quite evident, as health care needs to save money may be at odds with patients’ needs for access. Furthermore, health care institutions may attempt to limit patients’ use of ED, urgent care, office, and clinic services to be cost-effective or to use less costly (and less qualified) staff in the telehealth process, thereby reducing safety [10,19].

In the United States, evidence-based electronic guidelines have not yet emerged. No telehealth-based professional organization yet exists. Some agencies have developed regulations, including the Utilization Review Accreditation Commission (URAC) [20], the American Academy of Ambulatory Care Nursing [21], the American College of Emergency Physicians (ACEP) [22], the American Nurses Association (ANA) [23], the Emergency Nurses Association [24], and the North American Nursing Diagnosis Association (NANDA) [25]. Inadequately designed technology can lead to unintended consequences, while field testing may not be adequate [26,27]. The ACEP [22] has developed descriptions and broad classifications of emergent to acute symptom patterns for on-site triage. There is a need for a similar classification for teletriage.

Clearly, we are in early days in telehealth research, with the need to define meaningful measures for safe outcomes (Table 1).

**Table 1:** Safe outcomes.

Outcome	Description
AR^a^	A timely, safe disposition: right place, right time, for the right reason. ARs are considered safe.
OR^b^	A referral deemed (retrospectively) by some to be unnecessary at the time and place initially recommended. ORs are likely safe but not cost-effective.
UR^c^	A referral to a lower level of care than is safe or timely, often resulting in a delay in care. URs have the potential to cause or result in patient harm [8].

^a^AR: appropriate referral.

^b^OR: overreferral.

^c^UR: underreferral.

Controversies have emerged in relation to referrals (outcomes). Both appropriate referrals (ARs) and overreferrals (ORs) are considered safe, but ORs are not cost-effective—a less desirable outcome. Some experts suspect that doctors of medicine (MDs) are reluctant to define safety or to criticize other MDs’/researchers’ work.

Without a consensus on safe outcomes that are evidence based, it will be difficult for the industry to make meaningful progress toward the goal of safety. Research on meaningful safe outcomes is needed. We chose to discuss teletriage safety for several reasons: patient safety and safe practice are important topics, and teletriage has a high potential for error.

Teletriage involves making medical decisions under conditions of uncertainty and urgency. Teletriage has also conflicting goals: the institutions’ need to control costs (especially in the United States), while also ensuring patient access to care in a safe, timely manner. Furthermore, this underregulated subspecialty lacks adequate research on system safety.

Our review and analysis present a glimpse of current safety through analyzing 2 developed representative systems in 2 countries: Israel (Clalit Health Services) and the United States (Redwood Healthcare Plan [RHP]). We examined each system to learn how developed each system is and to explore the elements that might influence safe practice and patient outcomes.

This might be the first study to review and compare 2 formal teletriage systems. Both authors have performed triage in formal systems and taught and consulted in telehealth for a combined 50 years. Teletriage was the focus of this study. We focused on urgent, time-sensitive calls from patients or their families regarding acute or worrisome symptoms. We believe the telehealth industry and telemedicine can benefit from our findings.

## Methods

### Study Design

This study included 2 parts:

- A narrative review of the literature: studies describing nurses’ and physicians’ teletriage systems from the United States and Israel
- Qualitative assessment, including interviews with several physicians (Israel) and 1 nurse (the United States)

### Part 1: Narrative Review

Both key authors of this paper have practiced in the field of telehealth; thus, they have a reality-based perspective on the subspecialty.

We restricted our review to system features (structure, process, and outcomes) to provide a more orderly review in this variable and broad field. In this narrative review, we discussed the various facets of and challenges in teletriage, with special focus on the United States and Israel, which serve as representatives for teletriage for nurses and physicians, respectively.

#### Search Terms

Using the following key search terms, we searched PubMed, Medline, and Google Scholar for papers relevant to this review: Telephone Triage AND Teletriage AND Telehealth AND Telemedicine AND Telecare (and Tele-Triage); + Safety, + Systems, + Physician-led, + Nurse-led. + System Error, + Human Error.

#### Selection Criteria

It was essential that we study developed systems, because even today, US telehealth practice is still typically unregulated—variously devoid of complete or evidence-based components, such as guidelines, formal documentation, qualified staff, clinical training, and standards—in many office, clinic, ED, and ambulatory care settings.

For our critical analysis, we focused on the best examples of current practice: large, formal clinical call centers or HMOs. These services in the United States provide 24–7 clinical call coverage.

We narrowed our review of the literature to studies that focused either directly or generally on teletriage safety. See Multimedia Appendix 2 for criteria for selecting papers. Only English language publications that were published in scholarly journals or organizations between 1970 and 2023 were included. All types of papers were considered, including original papers, reports of randomized clinical trials, observational studies, and editorials or essays by key opinion leaders.

A summary of early research (1977 to the 1990s) focused mostly on the physician practice of teletriage. A recent review (the 1990s to 2022) summarized and critiqued teletriage safety research.

### Part 2: Qualitative Assessment

Using a qualitative approach, we conducted interviews with 15 representative physicians who worked in a pediatric teletrige service (Clalit Health Services) in Israel. In addition, we interviewed 1 nurse who worked in a nurse teltriage service in the United States.

To obtain their subjective perspectives on maintaining patient safety in this setting, the physicians were asked about factors that may have impacted their reaching a “correct” diagnosis and deciding on reasonable and appropriate treatment.

To gather detailed and accurate information that would accurately reflect the participants’ subjective experiences, we used a semistructured qualitative study (SSQS) technique in this study. Participants’ replies were evaluated and analyzed thematically when themes were found.

The use of open-ended questions, which gave the study its qualitative quality, allowed participants to candidly discuss the challenges they encounter in teletriage settings and the strategies they use to ensure patient safety.

The research complied with the Standards for Qualitative Research (SRQR) items [28]. We examined the responses using qualitative content analysis, which is a systematic procedure for collecting and analyzing qualitative data. Using a consistent set of codes to group texts with comparable content and creating themes and subcategories within themes from participant replies, this technique aims to “answer questions such as what, why, and how, and the common patterns in the data are searched for” [29].

### Ethical Considerations

Informed consent was obtained from the physicians and nurse participating in the qualitative section of the study. All necessary approvals for this study were obtained from the Ethics Committees of Clalit Health Services and the University of Haifa (approval numbers 0031-16COM2 and 458/16, respectively).

## Results

### Telemedicine and Teletriage Growth Surge During the COVID-19 Pandemic

Telemedicine, or the use of digital and remote medical technologies to connect patients and caregivers, has become the hottest and most talked-about area of technology, thanks to the COVID-19 pandemic. The influence of the pandemic on the area of telemedicine worldwide is best summarized by the *New York Times* headline “10 Years of Change in One Week: Telemedicine on Fast Track” dated April 20, 2020.

COVID-19 plagued the world for most of 2020, posing a serious threat to public health. Although many health organizations were primarily focused on combating the immediate effects of COVID-19, maintaining basic and vital therapeutic services was equally important. Initial responses in many nations included clinic closures and the suspension of all noncritical medical services [30,31].

Telemedicine provides ongoing medical care, while maintaining strict social distancing. To reduce their exposure to others and still obtain medical care, patients at risk may benefit from staying at home. As a result, it is not surprising that health care systems worldwide are turning to telemedicine, which has led to an exponential surge in its use as opposed to a previously slow uptake of the novel practice [32,33]. Thus, because of the COVID-19 pandemic, teletriage services have been implemented more frequently [34].

The benefits of teletriage during the COVID-19 pandemic have been described in recent studies; these studies show that this technique removes face-to-face contact, lowers the danger of exposure for medical personnel and other patients, and conserves scarce resources. Results suggest that more investigation is needed to ascertain how teletriage affects clinical outcomes, expenditures, and the use of follow-up care [35,36].

Although the COVID-19 pandemic has fueled the awareness and growth of technology and televisits, which are a convenience and infection control, the COVID-19 period has not made teletriage systems safer. It has made technology proliferate explosively.

### Teletriage: First Point of Access to Care

Patients call advice lines for a reason. They want to know whether their symptoms are urgent. Clinical decision makers assess the symptoms, estimate the urgency, triage the symptoms, and advise when, where, and why the patient should be seen. Teletriage is designed specifically for this purpose—estimating symptom urgency and triage to ensure timely access to care. On the continuum of care, teletriage can now be acknowledged as the entry point to clinical care. It legitimately qualifies as “prehospital care” [8].

The primary function of teletriage is the assessment and management of symptoms by telephone, which also calls for expert judgment, clinical evaluation, and proactive information gathering from the patient [6,37].

According to researchers, nurses estimate and rule out symptom urgency to determine a disposition by using pattern recognition. “Telephonic medical diagnosis of patients’ problems” is what telephone medicine, as practiced by doctors, is defined as [15,38].

### Teletriage System Safety

The task in teletriage is to safely assess symptoms, estimate the urgency, and triage the symptoms presented remotely and then advise a disposition (time and place) for them to be further evaluated. The goal is to “make good decisions under conditions of uncertainty and urgency” to avoid the risk of delay in care, diagnosis, or treatment. Compared to in-person consultations, teletriage is a complex activity that entails certain inherent dangers because there is no visual contact and no nonverbal communication [39-41].

While performing teletriage, nurses must rely on audio signals rather than visual ones, although patients can speak about their symptoms using different terms. The ability of clinicians to communicate effectively is crucial, but there are also several other abilities that must be present, including the ability to recognize verbal cues, concentrate on obtaining a focused history, and understand the importance of having proper documentation [14,42,43].

Other characteristics of after-hours care that could pose risks include a high patient call volume, a variety of clinical conditions presented, the likelihood of urgent conditions being present, unknown patients, knowledge gaps regarding patients’ medical histories, and the potential for information transfer discontinuity. Concerns have been raised because teletriage might compromise patient safety [44-48].

Regarding the reliability and safety of teletriage services, several recent studies have produced contradictory findings. Some studies were pessimistic, reporting that patient safety is frequently jeopardized by teletriage decisions [49]; service providers do not always forward the case to the on-call physician, when necessary [50]; and only a small number of diagnostic and therapeutic choices made during teletriage consultations offer the same level of health care as in-person conversations [51]. Inadequate visual cues that help doctors identify patients in acute condition were indicated as patient safety hazards in a study using teletriage [40].

However, more reassuring findings have been reported by other studies on the safety of teletriage systems. For instance, Blank et al [52] reviewed studies in which telephone counsel was contrasted with professional advice that is thought to be acceptable in that circumstance (ie, the “gold standard” of professional advice). The accuracy/appropriateness rate was 44%-98% in this review, with a median of 75%. Most decisions were appropriate according to a different study [14].

Concerning teletriage system effectiveness, the evidence also points to a variety of outcomes. According to certain studies, teletriage interventions, particularly for parents of small children and for older patients with chronic diseases, significantly reduce the number of emergency visits and readmissions [53,54]. Additionally, patients have stated that teletriage services have gained their trust and satisfaction. One study, however, found that a significant portion of patients who were directed to the ED using teletriage may have been treated elsewhere [55].

Based on a summary of several systematic reviews, when considered as a whole, the available research does not offer conclusive answers to queries concerning the standard of care delivered, the equity of access, costs, or outcomes in teletriage settings [18].

Growth alone in a new subspecialty will not guarantee safety. Developing a safe system is essential to any subspecialty, especially teletriage and telehealth. Defining the new subspecialty is one of the first challenges and sets the stage for transparency and, later, safety [14].

Even with the use of video and other technologies, remote symptom assessment is a uniquely risky task. Fraught with uncertainty, and many unknowns, teletriage is extremely time driven and time sensitive. A delay in care can be lethal if a required follow-up evaluation and treatment are not performed in a timely manner. In addition, teletriage is still in an underdeveloped state and lacks a reliable system. Finally, nurses and physicians perform this decision-making task under surprisingly difficult conditions [14,43-46].

Human factors in teletriage that challenge and possibly impair clinicians’ decision-making process are detailed in Textbox 1.

**Telehealth risks (human factors).**

- Inability to see patients (technology dependent)
- Ability to see but not to touch or gather patient vital signs (technology dependent)
- Extreme brevity of patient encounters (5-15 minutes)
- Incomplete or inaccurate information provided by patients
- Extensive sensory deprivation (endured by clinicians; technology dependent)
- Physical and cognitive demands imposed by high call volumes
- Potential for decision fatigue due to call volume and repetitive nature of the task [26,56]
- Clinicians often not knowing the patient, their education level, or their likelihood of compliance with advice
- A lack of structure (standardization of process and structure)
- Institutional pressures on clinicians to act as a gatekeeper rather than an access facilitator
- User-unfriendly electronic and paper guidelines

One way to avoid the risk of delays in care is to create a system. The Donabedian model [57-59] provides a framework for examining and evaluating health service quality. According to Donabedian [57-59], information about the quality of care can be drawn from 3 categories: structure, process, and outcomes.

Like other subspecialties, teletriage requires certain components to support safety. These components include standards (policies and procedures); sufficient numbers of qualified, experienced clinicians; specialized clinical training in medical decision-making; evidence-based, transparent, user-friendly guidelines; and electronic medical records (EMRs), audiotapes, or written documentation.

#### System Components: Evidence of a Duty to Care

Not surprisingly, in malpractice cases (when an error has occurred and a patient has been injured or has died), expert witnesses for the plaintiff always request tangible evidence of the system [9]:

- Guidelines used in the call (paper or electronic, eg, computerized decision support system [CDSS])
- Qualified experienced clinicians: résumés of nurses who managed the call, adequate numbers of clinicians
- Standards or policies and procedures, including job descriptions and qualifications
- Call center standards
- Actual call documentation: EMRs, paper form, or transcription of audiotaped calls
- Clinical teletriage training program materials

#### System Error

System error is thought to be the worst form of medical error [26]. Determining the effect of safety requires an examination of the problem of system error, defined as a failure of systems, processes, or conditions that are intended to prevent errors from occurring and that might lead people to make mistakes [60].

The Institute of Medicine (IOM) [60] has broadly defined system error as the “wrong match of plan” or the “failure to use any plan” to prevent error. For example, IOM research shows that the after-hours time, when no system in place, is especially risky in the United States [60]. In telehealth, complete systems (process or structure) are a first step toward reducing system error. Complete does not imply evidence-based or quality systems, however [8].

#### Malpractice in Teletriage

When a patient is harmed through unsafe telepractice, a malpractice case ensues. The plaintiff’s expert witnesses request evidence of care for that event: all documents that provide evidence of an adequate system, as described before. Institutions that can produce evidence of care are more able to demonstrate fulfillment of the duty of due care.

Physician teletriage malpractice may be related to the lack of a basic, complete teletriage system [16,49,61-65]. Nurse teletriage malpractice may be related to both the lack of a complete system or practicing in a complete system made up of faulty components [6,8,44,66-68].

#### What Are Meaningful Outcome Measures?

“We don’t look for patterns of our recurrent mistakes, or devise and refine potential solutions for them. But we could, and that is the ultimate point” [69].

We know what error and near misses look like. However, we have not yet clearly defined what constitutes safe practice and outcomes. Many researchers define telehealth safety variably, based on medical consensus on a study-by-study basis. Research continues to focus on nonessential elements of the process or structure (ie, communications, type of practitioner, patient compliance, and satisfaction).

The unfortunate outcomes described in malpractice [70-73] serve as fragments of the larger picture—system error, the essential and underrecognized problem.

Historically, medicine and nursing adhere to the key obligation “First, do no harm.” Nonmaleficence, which is derived from the maxim, is one of the principal precepts of bioethics—a fundamental principle worldwide.

Currently, professional organizations, such as the ANA [23] and the American Medical Association (AMA), typically set standards to guide medical decision-making, ethical practice, and patient safety. Formal systems—evidence-based structures and methods and guidelines—support clinicians’ safe practice and promote safe outcomes. Such system components are evolving slowly.

### Safety Studies on US Teletriage

Research on the safety of teletriage systems, whether practiced by registered nurses (RNs) or MDs, is scarce [54].

#### Safety Research in the United States

Early studies examining the system structure and process provide a basis to inform research on system error. Although safety is often a topic of telehealth research, to the best of our knowledge, system error is still underresearched.

It is likely that the proprietary nature of telehealth technologies interferes with research on system safety. Telehealth trends make it difficult to achieve system transparency. The field urgently needs evidence-based CDSSs, EMRs, and other new technologies, such as features that provide feedback on outcomes to clinicians for the purpose of learning from their mistakes or successes.

In addition, CDSS, computerized decision-making system (CDMS) and EMR components, so fundamental to the clinical decision-making process, make it essential that these technologies be demonstrably and verifiably safe and effective. Questions remain about the safety of guideline technologies [74].

#### Early Research (1977-1990)

Early studies on teletriage focused on physician practice. Predictably, key demographic groups of frequent calls included infants and children, the elderly, and women. Topics also included categories of symptomatic calls and urgent situations: the sudden, rapid death of children, calls to the ED and poison centers, postpartum concerns, suicidal callers, and cases resulting in malpractice [9].

The first studies on remote telephone encounters often focused on problems that plagued physicians: strategies for reducing inappropriate after-hours calls, follow-up postdischarge calls, characteristics and perceptions of after-hours callers and high users (“frequent flyers”), call patterns, and dissatisfaction in pediatric practice. In general, US physicians were dissatisfied with the task of teletriage [9].

Research by Perrin and Goodman [75] marked the beginning of a change in how teletriage was practiced in the United States. The study compared the teletriage practices of pediatric nurse practitioners (PNPs) with those of pediatricians. Researchers found that PNPs are as safe and proficient as physicians, although PNPs take slightly more time to manage calls.

#### Research in 1990-2000

Research later focused on nurses’ safety: communication, close calls, malpractice claims, access, chest pain, the influence of after-hours calls, and clinical and nonclinical decision makers. Later, the first teletriage training manual for nurses was published [9]. Lephrohon and Patel [14] showed how nurses practicing teletriage made decisions, describing pattern recognition and estimation of urgency as key decision-making strategies.

#### Research in 2000-2023

In the 2000s, rudimentary systems emerged [8]. Research highlighted the field’s disorganization and lack of professional development [76]. Patient safety research was inconsistent and of variable quality, often commingling widely different clinical and nonclinical decision makers, intermingling terminology, and making unquestioned assumptions. Evidence-based studies were sparse.

A recently published systematic review [77] assessed the effectiveness of teletriage as one of these technologies during the COVID-19 pandemic. Studies investigating teletriage’s effect on patient safety, clinical outcomes, and patient satisfaction were included. The authors concluded that teletriage interventions reduce unnecessary visits, improve clinical outcomes, reduce mortality and injuries, increase patient satisfaction, reduce health care provider workload, improve access to primary care consultation, and increase patient safety and satisfaction.

In Multimedia Appendix 3, we describe a developed teletriage center in the United States and include an interview with a qualified nurse working in this call center. Throughout the interview, she describes her personal feelings and reflections. Table 2 describes the required education, key system components, decision-making strategies, and goals of both Israeli physicians and US nurses.

**Table 2:** Decision maker comparison: Israeli MDs^a^ and US RNs^b^.^c^

Decision maker	Minimum qualifications	System components	Decision-making strategies	Task objectives
Physician (autonomous, licensed clinician)	Doctorate level: 7 years of science-based clinical education and training + pediatrics specialty training for 4.5 years	Documentation: Regulation: state medical board clinical training, guidelines	Diagnosis Clinical judgment Critical thinking	Make a medical diagnosis. Identify and verify emergencies and urgencies.
Licensed nurse (autonomous, licensed clinician supported by a medically developed CDSS^d^)	Associate of arts (AA)/bachelor of science (BS)/master of science (MS)/doctor of nursing practice (DNP): 2-7 years science-based clinical education and training	≥3 components Guidelines: CDSS Documentation: EMRs^e^/audiotaping, clinical teletriage training Practice standards: American Academy of Ambulatory Care Nursing (AAACN) Call center standards: URAC^f^ Regulation: Board of Registered Nursing	Pattern recognition Clinical judgment Contextual information Nursing process Critical thinking	Identify and verify emergencies and urgencies. Estimate symptom urgency. Rule out symptom urgency. Interpret patient responses.

^a^MD: doctor of medicine.

^b^RN: registered nurse.

^c^Partially adapted from Wheeler [8], with permission.

^d^CDSS: computerized decision support system.

^e^EMR: electronic medical record.

^f^URAC: Utilization Review Accreditation Commission.

### Telemedicine and Teletriage in Israel

In Israel, most of the health care and social assistance is public, including health care, welfare, child support, and old age and disability benefits. The national mandatory statutory health insurance system used in Israel is based on the Bismarck model. Both designated and ordinary taxes are used to pay for it. All citizens are required to join 1 of the 4 health plans (also known as mutualities or sick funds). The health plans provide both insurance for their members and a public basket of services, either through operating their own services or entering into contracts with service providers [78-80]. All 4 health plans are fully computerized, and all doctors and most other health care providers use EMRs that either are directly linked to the central medical record of the health plan through the internet or comprise its whole internal system. Between all community services, there is practically complete clinical data sharing. Highly developed decision support systems help with these.

Each health plan has highly advanced personal health records that allow members to access their own medical data online. These data entail prescription drug purchases and visits to the doctor, as well as imaging, laboratory, and other diagnostic test findings. Most of this is presently available online and via a smartphone in at least 2 of the health plans. Based on medical data and protocols created by the health plans, these plans currently provide proactive warnings and reminders for their members. The doctors at Maccabi, the second-largest health plan, can view their computerized medical information using a smartphone [78].

In Israel, physicians typically provide for all telehealth services, referred to as telemedicine. The physician practice of medicine or telemedicine is a range of remote high-tech remote encounters. The Ministry of Health (MOH) in Israel has regulations that apply to telemedicine services. Telemedicine standards were released in 2012 and have since been revised, as necessary, for different medical specialties. The MOH [79] provided an update in 2019 that details requirements for providing medical care remotely.

Although the worldwide pandemic has significantly accelerated what appears to be the next digital medical revolution, Israel has long recognized the enormous potential of telemedicine and has made it a national priority by allocating significant resources, establishing pertinent regulations, and promoting partnerships between health organizations, research institutions, start-up businesses, and independent researchers.

“Digital Health as an Engine of Growth” is a national priority program that Israel declared in March 2018. By using the information and communication technologies that are readily available to the entire Israeli population, the Israeli MOH [80] has stated that it is its mission to “bring about a leap in the health system that will enable it to become sustainable, advanced, innovative, renewed, and constantly improving.” In other words, the opportunity to further implement and expand a variety of telemedicine solutions is created by the worldwide acceleration of technology development and the digital revolution. The realization of the significance of digital health for the efficiency of the health care system and the requirement to offer strategic, systemic, and all-encompassing solutions for the foreseeable future are embodied in this national priority program [80].

Israel benefits greatly from a mix of human resources, a sizable number of businesses engaged in the development of digital medicine, and a sizable investment in research and development (R&D). It is a leader in communications and cyber innovation, which is essential to the creation of cutting-edge digital medicine that will be used worldwide.

The conditions for the successful implementation of telemedicine in Israel are encouraging: the population has individual identification numbers, digital medical records are stored in sizable databases, all people have access to medical insurance, the standard of medicine is high, and communication technologies are of high quality and are widely available throughout the country [81].

In Israel, all health plans operate telemedicine services in one form or another. For administrative requirements with the clinic and the attending personal physician, they all permit online services. With each of them, the attending physician can also be reached via telephone or video call during clinic hours and sometimes even after hours.

Additionally, several of the health plans offer online pediatric and family services that primarily act as medical triage after working hours, throughout the evenings, nights, and weekends. The patients can use telephone or video calls and occasionally even submit images during the online consultations [82,83].

Some health plans have also begun using the TytoCare test device, which enables online physical assessment. During a digital visit, the equipment checks the patient’s heart rate, respiration, temperature, ears, throat, and skin lesions using a variety of medical devices. A few Israeli hospitals have already begun to offer telemedicine consultations, particularly for presurgery evaluations, follow-up care, genetic and dietitian consultations, and even remote rehabilitation.

The quality of the telemedicine service provided and its safety are now the 2 most important factors to consider. Some telemedicine promotion initiatives during the pandemic seem to be predicated on the idea that a sizable part of outpatient visits may be effectively managed remotely, and patients can be prioritized for telemedicine services without endangering their safety or the standard of care [84].

An Israeli study [85] emphasized the growth in telemedicine usage during the first COVID-19 lockdown in Israel, as well as the anticipated partial fall in usage following the pandemic’s end. As of May 2020, most Israeli pediatricians recommended that once the pandemic has passed, they return to in-person consultations and base their therapeutic judgments on frontal data rather than on data obtained through telemedicine contacts [85].

There are not many studies on the safety of telemedicine or teletriage services conducted in Israel. Haimi et al [84] examined the level of safety of a pediatric telemedicine service, paying particular attention to the accuracy of the diagnoses and the reasonability of judgments made by the online doctors. This service serves as a time-sensitive teletriage of spontaneous calls from parents about acute, worrisome symptoms of their children that require triage (symptom sorting). The study showed high levels of diagnosis accuracy (98.5%) and decision reasonableness (92%).

In addition to the literature review, using a qualitative study, we interviewed 15 physicians who had worked at the Clalit Pediatricians Online Service (a teletriage service) over the past 5 years [82-84,86]. Using a semistructured interview protocol form, we questioned the physicians about the difficulties and obstacles they face in the teletriage setting that may affect their capability in maintaining patient safety. In addition, they were asked about their perceptions of their capacity to uphold patient safety in this teletriage environment and, in particular, regarding elements that impacted their capacity to make reasonable decisions, determine the best course of action, and diagnose accurately, while upholding patient safety.

The physicians described several difficulties they face in the teletriage setting that may impact their ability to maintain patient safety [84]. The main factor was the difficulty to make a diagnosis from a distance due to the physician’s inability to perform a physical examination in the telemedicine setting. Additional factors were treating unfamiliar patients, working alone, working under stressful conditions, having technological difficulties, and having a moral conflict between their desire to please and provide parents with good service on the one hand and the wish to maintain good medical practices on the other. While describing the challenges they face the teletriage setting, the physicians described various techniques and tools that they use to ensure patient safety.

Using a thematic analysis, we used the participants’ replies to determine themes. These themes were compared with the original transcriptions to determine whether they accurately reflected the original data, guaranteeing a constant flow. The following themes were gleaned from the interviews with the 15 physicians:

- Use of intuition: Many physicians claimed to have used their intuition during the diagnostic process and frequently in relation to parents.

You learn to rely on your intuition … whether you feel that the parents understand what you are saying, or that in this case, your instructions won’t help.

There is adversity, especially regarding certain decisions—I am sometimes hesitant about what to do, since I'm alone, especially at nights, and have to rely a lot on my intuition.

- Expertise: Most medical professionals believe that their clinical expertise in pediatrics in general and in telemedicine in particular aids in their diagnostic and decision-making processes. The more experience a medical professional has in telemedicine, the more confident they feel.

During my first few days at work, I was afraid I would miss things or that there would be problems. After a while, however, I began to work with more confidence and less stress.

There are some difficult aspects. At first, I felt insecure, but over time I gained experience (even the ability to diagnose better than the face-to-face doctor)! Like diagnosing a child with diabetic ketoacidosis …

- Using protocols: Many physicians said they use protocols and rules of thumb when making decisions. Most also use the protocols that are generated for special circumstances. They believed this assisted them in maintaining patient safety. They were also conscious of potential biases in their thinking.

I use protocols. For example, head injuries among babies under the age of six months, or a high fever among babies younger than one month old. These make it easier to make a decision.

I use some rules of thumb. For instance, if a young boy is able to jump around, then he does not have appendicitis.

- Making shared decisions with parents: A few medical professionals reported talking to the parents of their patients about their opinions on the diagnostic process and potential treatment options.

I used to share my decision-making process with the parents. If there were several options, I would let the parents decide. In such a case, I depend on them.

I usually share, but I do not consult. I give my opinion and explain it, and only then do I wait for feedback.

- Using nonmedical factors: Most of the physicians agreed that they consider nonmedical considerations, in addition to medical factors when making decisions. Their opinions of the parents, particularly their level of comprehension, anxiety level, health literacy level, and the assurance that the parents will act appropriately if the child’s illness worsens, are the most important considerations. The family’s ability to access medical care was another crucial nonmedical element.

In addition to medical factors, the parents’ tone of voice and level of stress may affect my decision, even if it seems to be a simple diagnosis … Language is also a factor. For example, new immigrants do not always understand me, and I am therefore more prone to sending them to the ED …

Aside from the medical condition, the patient’s place of residency is also important. Living far from a medical care facility is a factor, and I will be more likely to consider an ED referral. In such cases, I also ask more questions about the availability of the doctor nearby.

You have to trust the parents' information and rely on them to follow the instructions correctly. If I feel that the chances of me being understood are poor (due to a lack of understanding or oversophistication on the part of the parents), I will refer them to the ED more easily.

- Additional techniques: The physicians schedule video conversations with the parents in cases of diagnostic doubt, ask them to send digital images, or schedule a follow-up call a few hours later.

If I needed additional information, I would arrange a video call or a follow-up call at a later time. Rarely would I consult with a senior physician.

Despite the difficulty making the decision, pictures and videos often compensate for the lack of a physical examination … In one case, I managed to correctly diagnose a child with intussusception!

Despite the difficulties and obstacles mentioned by online doctors [79], many of the physicians surveyed in this study reported having generally positive experiences with their telephone assessments and feeling confident in their ability to conduct thorough assessments and reach the right treatment decisions.

The key conclusions, with examples and comparisons between the 2 systems, are shown in Table 3.

**Table 3:** Key conclusions derived from the findings.

Key topics			Findings
**Specialized clinical training for teletriage tasks**			
	RHP^a^	The RHP does not provide formal specialized teletriage training for nurses. However, it requires formal training for its electronic algorithms*.* Physicians present lectures on various specialties for the nurses.	
	Clalit Health Services	Teletriage training for pediatricians is not available. The authors believe training would aid pediatricians in making safer decisions during online consultations.	
	Conclusion for both systems	Judging from the interviews with nurses and physicians, it appears that both systems’ clinical training is not adequate and formal training would be beneficial. Clinical training for any new subspecialty is an essential safety measure. Research has shown that clinical preparation has the potential to build confidence, improve performance, and reduce error, while improving morale [70-72,75,82,83].	
**Electronic algorithms and protocols**			
	RHP	With rare exception, the RHP requires nurses to follow and heavily rely on electronic algorithms in decision making. This raises the question of whether the RHP’s electronic algorithms function more as a CDMS^b^ than as a CDSS^c^ [73]. The nurse interviewed (Ms Finley) stated that the overreliance on algorithms discourages nurses’ critical thinking and dampens her initiative to perform a more thorough preliminary symptom assessment and to promote interpersonal interactions.	
	Clalit Health Services	The Clalit system provides several written protocols for certain clinical scenarios, and physicians are encouraged but not required to use them. In our qualitative interviews, many physicians said they used protocols and rules of thumb when making decisions.	
	Conclusion for both systems	For both nurses and physicians, guidelines are a key decision support tool. In addition, guideline quality (validity and reliability) requires evidence-based research—long overdue in this risk-prone field.	
**Documentation**			
	RHP	The RHP system provides 2 methods for call documentation, an audiotape recording and an electronic paper trail—a record of the patient-clinician encounter derived from a given guideline. However, the documentation output is limited to a patient’s yes/no responses to the algorithmic questions. The result is an anonymized history with few details or context specific to a given patient [26]. Finley stated that physicians who later evaluate patients on-site do not have a good sense of why the patients were advised to be seen urgently. The RHP later developed a new policy allowing nurses to use a free-text area to document a brief symptom history using standard questions to elicit more specific details and context. Quality assurance is further bolstered by audiotaping all calls for follow-up review.	
	Clalit Health Services	The Clalit system requires physicians to document calls, completed in the child’s medical file. As a result, the personal physician can view the online consultation during business hours. However, the language used in the documentation is completely up to the individual physician.	
	Conclusion for both systems	The RHP “paper trail” appears safer and more complete. However, the documented output appears to introduce confusion into on-site follow-up encounters. Clalit Health Services’ lack of standardized language requirement may interfere with communication and continuity of care—a professional principle. Both systems are inadequate and increase miscommunication—one of the most common, recurrent error in this field.	
**Clinical call center standards (policies and procedures): clinicians’ knowledge and experience**			
	RHP	According to Finley’s interview, the RHP appears to have no job requirements or job descriptions and according to its policy may hire inexperienced nurse graduates. New nursing graduates are a poor match for the medical decision-making task, which according to many experts, requires a minimum 5-year bedside experience.	
	Clalit Health Services	The Clalit system hires only certified pediatricians, even though their level of experience as pediatricians in general and as online physicians may vary greatly.	
	Conclusion for both systems	Experience is critical in decision-making. Both groups could benefit from improved standards for required experience and job qualifications.	
**Clinical call center standards (policies and procedures): call length (teletriage meeting duration)**			
	RHP	Although it is the customary role of management to develop call center policies and procedures (standards), at the RHP, staff nurses have developed a minimal number of standards. One is a maximum call length, while another is a closing reminder to callers to call back if symptoms worsen or change.	
	Clalit Health Services	The Clalit system does not place any constraints on session length. However, since physicians are paid “per consultation,” it may be an incentive to process calls quickly, although using the best medical decision.	
**Clinical call center standards (policies and procedures): patient outcome feedback**			
	RHP	RHP nurses operate in a vacuum regarding patient outcomes (follow-up diagnosis). Outcomes provide feedback and are a measure of patient safety. Feedback about one’s decisions is essential to improved practice and one of the strongest risk management measures available [87]. The rationale for not providing feedback to nurses is based on the Health Insurance Portability and Accountability Act (HIPAA). This federal law does not prevent US physicians’ access to patient outcomes, however.	
	Clalit Health Services	Clalit physicians have complete access to the outcomes of their calls. Learning of their mistakes or successes may improve their practice and safety.	
	Conclusion for both systems	Ignorance about outcomes of one’s decisions has never been shown to improve practice. Feedback mechanisms, known as planned error recovery, not only allow practitioners to learn the final diagnosis and thus improve their practice but also may improve guideline design and quality.	

^a^RHP: Redwood Healthcare Plan.

^b^CDMS: computerized decision-making system.

^c^CDSS: computerized decision support system.

## Discussion

### Principal Findings

This narrative review and analysis presented a glimpse into current teletriage safety by analyzing 2 established and representative systems in 2 countries: Israel and the United States. We examined each system to learn how developed each is, perform a comparative analysis of both systems’ safety, and explore the elements that might influence safe practice and patient outcomes.

In the initial stage, we carried out a thorough analysis of papers pertaining to patient safety in teletriage scenarios. Current research yields conflicting results regarding the dependability and security of teletriage systems. Although some critics claim that teletriage decisions frequently endanger patient safety [40,49-51], other research claims that using teletriage systems results in better safety outcomes [14,52].

We also analyzed a clinical call center of a large national US HMO based on the responses of a representative advice nurse to an interview (Multimedia Appendix 3), highlighting areas of risk that may contribute to system error [17]. We found that this representative system is still underdeveloped and lacks certain risk management elements. We based our conclusions on the interview, recent research, legal and risk management requirements related to the duty of due care, medical and nursing traditions, and existing subspecialty structures and processes.

In addition, we performed a qualitative study in which we interviewed 15 Israeli physicians working in a pediatric teletriage service in Israel, asking them about factors that affect their ability to maintain patient safety, while providing an accurate diagnosis, making appropriate decisions, and choosing the best course of action [83]. The physicians discussed the challenges they encounter in the telemedicine/teletriage context and the many strategies they use to arrive at the best diagnosis and course of care, protecting patient safety. These strategies include using their experience and intuition, using protocols generated for special clinical scenarios, making shared decisions with the patients (or their parents in the case of children), applying nonmedical criteria to aid in decision-making in situations where the medical data are ambiguous, and using more sophisticated tools (eg, video chats) when additional details are required. Many of the physicians surveyed in this study reported having generally positive experiences with their telephone assessments and feeling confident in their ability to conduct thorough assessments and make the best treatment decisions, despite the challenges and blockages described [82].

This study may be the first to examine and compare 2 official telehealth systems. For a combined 45-50 years, the 2 authors have performed triage in formal systems, taught, and provided consultation in the field of telehealth.

Teletriage, as stressed in this research, is the process of evaluating and prioritizing symptoms using telecommunication technologies. The main goal of teletriage is to assess and manage symptoms by telephone, which necessitates the use of professional judgment, clinical assessment, and proactive patient information gathering. The purpose of teletriage is to determine whether the needed on-site evaluation should take place and, if so, the venue and time. Teletriage involves clinical decision-making under remote and uncertain conditions. An overarching goal of teletriage safety is to avoid delays in care or diagnosis, which can cause patient harm.

Clinicians typically estimate the urgency of acute symptoms remotely and advise a disposition (triage level) for further medical diagnosis and treatment, as appropriate. The growth of teletriage services has accelerated due to the COVID-19 outbreak.

All types of health care delivery must consider safety, but with teletriage, this is both more crucial and challenging because acute symptoms may be time sensitive. Delay in care and diagnosis can result in harm to patients. Since there is no visible contact or nonverbal communication during teletriage, it is a more complicated activity than in-person consultations and it has certain inherent risks. The rapid pace of telehealth’s growth creates urgency in identifying safe systems to guide developers and clinicians about needed improvement. Establishing a system is a key strategy to reduce the possibility of delay in care and diagnosis.

In the United States and internationally, one way to be cost-effective is to use the least paid person who can *safely* do the job—an RN. Internationally, nurses have traditionally performed this task since the late 1980s. Early studies found that nurses are a safe substitute for physicians [14,73]. Thus, although physicians initially performed this task, they later delegated it to nurses.

Health care institutions historically provide standard features to support nurses and to enhance safety (subspecialty clinical training, standards, and documentation). In the case of teletriage, guidelines are typically written by physicians, similar to standing orders. These components provide a structure and process for this subspecialty and underpin safe practice.

An evolving subspecialty, even after 50 years, teletriage appears misunderstood and neglected. System error is thought to be a result of the absence or inadequacy of systems. In malpractice cases, expert witnesses for the patient or their family request evidence of the duty of due care. Typically, this evidence comprises documents: call documentation, guidelines used, clinical training materials, policies, and procedures (standards), including written job descriptions and qualifications.

Clearly, this analysis must acknowledge that contexts of the institutions described here differ in terms of respective health care systems and decision makers’ clinical qualifications. The US health care system, and teletriage in particular, is plagued by disparate, competing forces: institutional cost containment, the need for professional standards, and diverging technological goals—the emphasis of speed over safety. This scenario requires better risk management.

Israel has universal health care, which appears to act differently. Physicians’ depth and breadth of education and clinical training are superior to those of nurses. The US health care system compensates for this difference by providing more structure in the form of guidelines—typically developed collaboratively by physicians and software engineers. Physicians are not actual users of the guidelines that nurses are required to use.

Another variable is that of the populations served. Clalit pediatricians serve the needs of a diverse but still circumscribed pediatric population, whereas RHP nurses serve a broad, diverse population in terms of age range, symptom presentation, and diversity. This is a large order for nurses to manage and calls for a robust structure and process.

Finally, both RHP and Clalit systems share a common problem: incomplete systems of variable quality. The Clalit system’s safety appears to rely on physician decision-making expertise, where standards, guidelines, and training are not that strong. The RHP may appear more complete. Safety may hinge on physician-developed electronic guidelines. Standards and training appear piecemeal or added as an afterthought. Without a meaningful, evidence-based structure and process in teletriage, quality (including safety) is at risk [18,58]. If establishing a system is a strategy to reduce possible error, then both systems could benefit from similar improvements.

Even if expert-level physicians require a less robust system, it appears that both physicians and nurses could benefit from specialized clinical training. In addition, consistent feedback regarding patient outcomes, known as planned error recovery—an essential error reduction strategy—promotes a method to self-check or to double-check another person’s work [87].

Teletriage electronic algorithms must be evidence based. These guidelines are typically collaboratively developed by physicians and software developers. Nurses are required to use them, whereas physicians rarely use such tools.

Our narrative review and in-person interviews with physicians and a nurse about their experiences working in teletriage settings yielded several key findings, including the absence of specific formal training for the medical personnel working in teletriage; problematic protocols in particular clinical scenarios that, although not always available for all scenarios, are of low quality and do not allow for flexibility and agility, when needed; problematic documentation (mainly in nurse teletriage); inadequate experience and knowledge of the personnel who must make decisions in the face of uncertainty and urgency; limitations on the duration of calls or compensation based on the number of calls (which incentivizes personnel to conclude sessions promptly); and unsuitable feedback mechanisms that prevent personnel from understanding what transpired with patients and from learning from errors.

Drawing from our individual findings, the essential elements of teletriage are:

- Specialized clinical training for teletriage tasks
- Electronic algorithms and protocols
- Documentation
- Clinical call center standards: clinicians’ knowledge and experience, call length (teletriage meeting duration), patient outcome feedback

### Limitations

As with any narrative evaluation, selection bias cannot be completely ignored, even if this narrative analysis of the current literature was quite extensive and comprehensive and included a qualitative assessment of physicians and a nurse working in a teletriage setting.

### Conclusion

Like other subspecialties, teletriage necessitates several elements to support safety, including qualified, experienced clinicians in sufficient numbers; specialized clinical training in medical decision-making; evidence-based, open, and approachable guidelines; and EMRs, audiotapes or written documentation, and standards (policies and procedures).

Fostering teletriage patient safety can be accomplished by taking the following general steps to improve MD and nurse practice in both Israel’s and the United States’ clinical call systems:

- Adequate training: Providers must receive adequate training to properly monitor and provide telehealth services. This includes knowledge of the systems being used, as well as familiarity with medical terminology and protocol.
- Regulation of telecommunication devices and systems: Providers must be aware of the regulations and requirements for the telecommunication devices and systems they use. This includes ensuring that the equipment is in good working order and adheres to all safety and security regulations.
- Appropriate patient population: Telehealth services should only be used to treat patients who are stable and not at risk for an immediate life-threatening event. This will help ensure patient safety and avoid unwanted outcomes.
- Careful monitoring: In appropriate consultations, when needed, providers must carefully monitor patients and document any changes in their condition. This will help ensure that any changes or issues are addressed quickly and appropriately. Typically, nurses do not perform this task; in Israel, this is the role of the physician, for example, by using devices such as TytoCare.
- Quality assurance: Quality assurance protocols must be in place to ensure the accuracy and effectiveness of providers’ services. This includes regularly reviewing documentation and providing feedback on any services deemed inadequate.
- Follow-up care: Providers must ensure that any patient receiving telehealth services receives follow-up care. This can include referrals to specialists or any other services needed to address any health concerns. Typically, nurses do not perform this task; in Israel, this is the role of the physician.
- Evidence-based studies of systems and safety: Misguided researchers unfamiliar with the triage task have produced confusing, misleading studies. Research that nibbles around the edges of the problem (patient or clinician satisfaction, clinician stress levels and attitudes, nonclinician practice) fails to address the core problem—system error. The telehealth industry requires long-overdue evidence-based outcome studies that meaningfully demonstrate the structures and processes that inform and strengthen safety.

## Appendix

Multimedia Appendix 1 Definitions and terminology.

Multimedia Appendix 2 Paper selection criteria.

Multimedia Appendix 3 Case study from the United States.

## Abbreviations

ACEP: American College of Emergency Physicians
ANA: American Nurses Association
AR: appropriate referral
CDMS: computerized decision-making system
CDSS: computerized decision support system
ED: emergency department
EMR: electronic medical record
HMO: health maintenance organization
MD: doctor of medicine
MOH: Ministry of Health
OR: overreferral
PNP: pediatric nurse practitioner
RHP: Redwood Healthcare Plan
RN: registered nurse
UR: underreferral
URAC: Utilization Review Accreditation Commission

## Data Availability

All data analyzed during this study are included in this published paper and its supplementary information files. Additional data sets generated during this study are available from the corresponding author upon reasonable request.
