# Supplementary material for: Safety in Teletriage by Nurses and Physicians in the United States and Israel: Narrative Review and Qualitative Study
Source: JMIR Hum Factors. 2024 Mar 25;11:e50676. doi: 10.2196/50676 (PMC11002740; doi:10.2196/50676)
Supplement: Multimedia Appendix 1 [file humanfactors_v11i1e50676_app1.docx]

**Appendix 1: Definitions and terminology**

**Defining Telehealth**

An internet search of the term “**telehealth**” demonstrates how the industry intermingles definitions. The search will produce results such as telemedicine, telephone triage, telehealth, nurse triage, phone triage and televisits and other forms of high-tech remote encounters in healthcare. The term telehealth has come to stand for both the industry-at-large, but also serves as a form of remote, virtual care, substituting for an on-site visit.

By the year 2020, telehealth already had many facets and subsets –Including telemedicine and telemonitoring and management of chronically ill patient groups, during the COVID pandemic, face to face visits meant possible contagion for patient and clinician alike. The growth of telehealth and televisits was explosive. Remote encounters became essential.

It is true that both televisits and teletriage share many things in common. Both are remote encounters. However, televisits are pre-*scheduled encounters* about *non-acute* problems. **Teletriage** involves unscheduled encounters about *acute patient symptoms* that require rapid assessment, urgency estimation and triage for further evaluation and diagnosis.

It is true that both televisits and teletriage share several in common they are both remote encounters, and eventually, high-tech services. However, their purposes are different. **Televisits** (pre-*scheduled appointments or virtual visits* for *non-acute* problems) are unlike teletriage (time-sensitive, urgent encounters for rapid assessment, estimation of symptom urgency and triage of *acute patient symptoms* that require safe, timely dispositions).

In the interests of clarity and transparency, we offer a list of terminology that is useful in discussions of the field and practice of telehealth:

- **Telehealth Industry**: is the distribution of health-related services and information via electronic information and telecommunication technologies. It allows long-distance patient and clinician contact, care, advice, reminders, education, intervention, monitoring, and remote admissions.
- **Telehealth**: Umbrella term for the new commercial/professional industry including all forms of high-tech remote encounters
- **Telehealth Practice**: Real-time remote encounters via video encounters between patients and clinicians.
- **Telemedicine:** is the practice of medicine by remote means. **Telemedicine** usually refers to physician practice of medicine in all forms of high-tech remote encounters.
- **Virtual visit or Televisit** (sometimes called Telehealth visit): are defined as a remote, pre-scheduled encounters for non-urgent symptoms using a range of technologies. It is a substitute for an on-site non-urgent visit. Substitution of a remote follow-up or non-urgent encounter for on-site encounter typically requiring clinical (MD or RN) assessment.
- **Teletriage**:  Remote, brief, urgent, unscheduled patient initiated clinical encounters to triage urgent symptoms utilizing a range of technologies. It includes all forms of high-tech remote encounters requiring clinical (MD or RN) assessment, disposition and triage of acute patient symptoms.
- **Telephone triage**:  remote telephone encounters requiring clinical (MD or RN) assessment, disposition and triage of acute patient symptoms. Telephone triage is the pre-curser of, will be subsumed by teletriage and telehealth.
- **Telemonitoring**: “Medical telemonitoring is a continuous or non-continuous monitoring process that allows a healthcare professional to remotely interpret the data necessary for a patient’s medical follow up and, if necessary, make decisions regarding the patient’s state of health”. Science Direct
- **Appropriate**: suitable or proper in the circumstance.
- **Computerized Decision Support Systems (CDSS):** Expert software systems that remind experienced decision makers of information to consider that s/he once knew but may have forgotten.
- **Computerized Decision-Making Systems (CDMS):** Expert software systems that allow an unqualified person to make a decision that is beyond his/her level of clinical training and experience.
- **Error**: An umbrella term that includes human error,

assessment and communications failures and under referrals.

- **Malpractice**: The term “malpractice” is specifically

related to professional negligence and is committed by a professional. In effect, professionals are held to a higher standard than non- professionals.

- **Negligence**: Failure to provide due care to patient.
- **Timely**: Coming early or at the right time.
- **Vicarious Liability**: Liability on the part of employers,

who become accountable for the negligence of an

employee.

Partially adapted from Safety of Clinical and Non-Clinical Decision Makers in Telephone Triage: A Narrative Review March 2015 [Journal of Telemedicine and Telecare](https://www.researchgate.net/publication/journal/Journal-of-Telemedicine-and-Telecare-1758-1109) 21(6), with permission.

**ADDITIONAL READING**

Gladwell, M. (2005). **Blink: the power of thinking without thinking.** Little, Brown & Co.,NY. <https://www.amazon.com/Blink-Power-Thinking-Without/dp/0316010669>

- Hirsh DA, Simon HK, Massey R, Thornton L, Simon JE. **The host hospital 24-hour underreferral rate: an automated measure of call-center safety.** *Pediatrics* 2007; **119:1139**-44

Kempe A, Bunik M, Ellis J, et al. **How safe is triage by an after-hours telephone call center?** *Pediatrics* 2006; **118:457**-63

- **NANDA International nursing knowledge association.** (2019) http://www.nanda.org/nanda-iresources/glossary-of-terms/National Council of State Boards of Nursing. (2014). Position Paper on Telehealth Nursing Practice <https://www.ncsbn.org/3847.htm>
- Reason J. (2000). **Human Error: Models and management.** BMJ. Mar 18; 320(7237):768-770. <https://www.bmj.com/content/320/7237/768>
- Reisman AB, Brown K. E. (2005). **Preventing communication errors in telephone medicine: A case-based approach.** Journal of general internal medicine. Oct; 20:959-63 <https://www.ncbi.nlm.nih.gov/pubmed/16191150>
- Smith, R. (2005). **Telephone Triage Risk Management,** TeleTriage Systems Continuing Education Course, teletriage.com TeleTriage Systems Publishers, San Anselmo, CE
- Wheeler, S. Q, Windt, J. (1993). **Telephone Triage: Theory, Practice and Protocol Development.** TeleTriage Publishers, San Anselmo, CA. https://teletriage.com/telephone-triagetraining/consulting/books/theory-/practice-and-protocol-development/
- Wheeler et al. (2015). **Safety of Clinical and Non-Clinical Decision Makers in Telephone Triage: A Narrative Review**, Journal of Telemedicine and Telecare. <https://www.ncbi.nlm.nih.gov/pubmed/25761468>
- Wheeler. S.Q. (1989). ED telephone triage: lessons learned from unusual calls, J Emerg Nursing <https://pubmed.ncbi.nlm.nih.gov/2687540/> 1989 Nov-Dec;15(6):481-7.   PMID: 2687540
