# Supplementary material for: Safety in Teletriage by Nurses and Physicians in the United States and Israel: Narrative Review and Qualitative Study
Source: JMIR Hum Factors. 2024 Mar 25;11:e50676. doi: 10.2196/50676 (PMC11002740; doi:10.2196/50676)
Supplement: Multimedia Appendix 2 [file humanfactors_v11i1e50676_app2.docx]

|  | **Inclusion** | **Exclusion** |
| --- | --- | --- |
| **Technology** | Live virtual encounters via Telephone or Video between Patients at home and Clinicians, regarding acute symptoms for the purpose of symptoms assessment, urgency estimation and triage and timely access to the appropriate level of care for further evaluation and treatment. | Asynchronous Video (AKA Store-and-Forward).  Remote Patient Monitoring (RPM).  Mobile Health (mHealth)  Scheduled non-urgent telehealth virtual visits: Email, messaging, texting, medical applications, telemonitoring, teleconference, other. |
| **Staff** | Clinicians: Physicians or Registered nurses. | Emergency Medical Technician, Paramedic, LVN, LPN, Medical Assistants, Physician Assistants, Nurse Practitioners |
| **Decision making Task** | MD: Medical diagnosis of Acute, symptom-based calls  RN: Pattern recognition (Lephrohon et al. 1995) of acute, symptom-based calls  RN: Estimation of urgency & risk of acute, symptom-based calls | Chronic Disease management  Poison control, crisis hotlines, health education, mental health counseling, referrals to specialists  Routine follow up appointment calls, medication refills |
| **Structure**  System Component | Component Presence Only  Years of experience  Clinical Training in Teletriage  Recording calls: Paper or Electronic Guideline (CDSS) Audio recording  Paper/ Electronic Documentation (EMR)  Standards Policies or Procedures | Component Quality:  CDMS quality  EMR quality  Clinical training quality  Standards or Policies and Procedures |
| Setting | Call Centers: clinical call center, Nurses and Physicians taking call from unknown remote site.  Emergency Department  Medical office, clinic, or home office |  |
| Outcome Measure or Safety | Appropriate Referral: Timely Emergency Department (ED), Urgent Care or Office Visit  Under referral: Referrals resulting in actual or potential harm, error, mistake, harm, injury, unanticipated hospitalization or ED visit, death. | Patient self-referral to ED or Urgent Care  Satisfaction of Patient, Clinician or Call handler.  Cost savings, Return on Investment (ROI)  Pt self-referrals to ED/Urgent Care |
| Time Frame | All Hours (Office and After Hours): 24/7/365 |  |
| Study Designs | International, reviewed, Observational, quantitative and comparative studies 2010- 2023 In English language. | Unclear results; commingled clinicians and non-clinicians; commingled groups other than two selected.  No abstract or full text  Editorials  Letters to Editor |

**Appendix 2: Article selection criteria**
