# Supplementary material for: Safety in Teletriage by Nurses and Physicians in the United States and Israel: Narrative Review and Qualitative Study
Source: JMIR Hum Factors. 2024 Mar 25;11:e50676. doi: 10.2196/50676 (PMC11002740; doi:10.2196/50676)
Supplement: Multimedia Appendix 3 [file humanfactors_v11i1e50676_app3.docx]

**Appendix 3: Example of a Teletriage system in the US (Interview)**

In this descriptive analysis and comparison, the goal was to paint as complete and contextual a picture of a U.S.- based system (the healthcare system and its clinical call center and the populations served, as well as the representative’ nurse’s perspective) to compare it with a similar Israeli system. All names of persons and institutions in this article are fictitious.

**Elizabeth Finley**, RN, BSN, is a seasoned nurse with 20 years of experience in the Intensive Care Unit, Emergency Department (ED), Medical-Surgical Unit, Post Anesthesia Care Unit, and the last five years in the call center. The sidebar describes her personal feelings and reflections. In addition, her observations of the call center also appear in parentheses throughout the article.

**OVERVIEW**

In contrast with Israel’s universal healthcare, the United States (U.S.) healthcare system resembles a patchwork of private and public healthcare services. **Redwood Healthcare System** (**RHS**) represents a managed care system – a “prepaid health plan that provides care through a network of providers under a fixed budget and managed costs”. RHS provides a remote access to advice nurses as a patient healthcare benefit for patients calling about new, worrisome, or serious symptoms. Clinical call centers are intended to provide access to clinicians, while at the same time reducing inappropriate (and costly) visits to the clinic, office, Urgent Care or ED.

The overall goal of teletriage is to help the larger healthcare system run more cost effectively. Although occasionally viewed as a form of gatekeeping, teletriage nurses typically view the work as facilitating patient access to care. Calls initialed by patients from home are the first contact (pre-hospital) in the Continuum of care.

Beginning in the1970’s, RHS provided informal telephone triage services from clinics and office settings. In the 1990’s (Wheeler, 2015) centralized clinical call centers emerged. The call center is staffed by nurses, and operates 24 hours a day, seven days a week. It serves patients across wide regions and takes millions of calls annually.

**CALL CENTER DESCRIPTION**

Patients typically call from home, and nurses typically take calls from clinical call center. Initially, few nurses worked from home. When the COVID pandemic emerged, the Nurses Union mandated working from home as a safer option for staff, and video and phone technologies were rapidly integrated into the task. Almost overnight, telehealth and teletriage became an essential feature of healthcare. This hybrid work setting will likely remain so for the foreseeable future.

**Call Center Setting and Staffing**

The physical space is a large room with cubicles. Between 1 to 200 staff may work there at any given time. Management “staffs to the season”, utilizing an algorithm based the previous years’ staffing patterns.

**Workload and Call Volume** Nurses are required to managed 6 calls per hour. (+/- 10 minutes per call). If wait times become too long due to high call volume, the call center manager steps in and answers calls. (Finley noted that although staffing adequately matched to the call volume, typically, coverage did not allow for socialization of co-workers – like taking breaks or meeting for lunch. The work felt isolative).

**Work Week** Many nurses worked a minimum of 24 hours per week to be eligible for benefits. (Finley noted that the job was lucrative, with overtime being the most lucrative.)

**Job Description/Job Experience/Qualifications**

When she was hired, Finley did not recall seeing a written job description, required experience, qualifications, or interpersonal skills. Finley described her co-worker's clinical education as ranging from Associate to Master’s degrees. Some managers hired new nursing school graduates with little bedside or decision-making experience. Finley speculated whether management over-relied on algorithms as decision *making* rather than decision *support* tools. A perceived overdependence on technology appeared to outweigh the need to hire experienced nurses, as in the case of new graduates.

COMMENT: Some experts believe that teletriage work requires a minimum of three to five years bedside experience (qualification in critical thinking and clinical decision-making).

**POPULATION DESCRIPTION**

Finley described the population in response to the author’s community assessment questionnaire. Nurses in RHS call center serve a uniquely challenging population – both broad and diverse. Calls come from patients that range widely in age, socio-economic status, education and literacy levels, language and ethnicity. The settings range from inner city to rural, or suburbs to traveling patients calling from overseas international sites or newly disembarking from cruise ships.

In general, the population is composed of healthy young adults and families. Age range is wide -- encompassing infants to elderly -- pediatric, school age, women’s health, to geriatric. Symptoms cover a wide gamut – from “chest pain to chicken pox”, as well as behavioral (i.e., emotional, or psychological) problems and trauma. Environmental dangers may include nearby refineries, forest fires, chemical spills, and occasional outbreaks of communicable diseases.

Endemic diseases include Sickle Cell Disease, HIV, High risk mothers/babies, and other diseases. Cultural/Religious Factors and Resources include religious practices (diet, gender-based attitudes, religious prohibitions - Muslim and patriarchal cultures).

Socioeconomically, patients range from illiterate working poor to highly educated and wealthy. Finley adds that consumer awareness of patient rights, health options and informed consent is quite high. Occasional non-members of the RHS health plan contact the call center. Per RHS policy, these patients must be referred to the nearest ED (Emergency Department).

Finley notes that many calls concern Alcohol and/or Drug abuse, where trust is key between caller and clinicians. Nurses often refer Patients to their Primary Care Physician as well as multiple support groups and hotlines.

COMMENT: The diversity of RHS population likely makes the work more stressful and demanding (and managements’ expectations higher) in comparison to an advice service specialized for OB/Gyn, Orthopedics or Cardiovascular populations, etc.

**Clinical training:** Since the 1980’s a variety of *clinical* training offerings for teletriage have become widely available (online, or on-site classes, continuing education programs, workbooks, audiotaped case studies, training manuals, workshops, and conferences). Training quality and consistent principles is unknown.

RHS had no formal clinical training program but required a month-long training in software operation. Occasionally, RHS Physicians presented Informal lectures on certain specialties. (Finley stressed her strong desire for, and the importance of, more clinical training.)

**Electronic Algorithms:** A large Health Information Technology company developed the algorithms in collaboration with RHS clinical staff. The Algorithms number in the hundreds; nurses revise the algorithms.

(Finley noted that software developers designed algorithms to reflect the circumstances of middle- to high-income populations. This approach assumed that callers were literate, had access to a car, money for medications and other resources. For low-income, low literacy, high risk populations, this was not the case. This meant that low-income patients with non-urgent (but treatable) symptoms (cellulitis, possible dehydration), required referrals to the ED (considered the venue of last resort) with the costly and unwelcome prospect of ED overcrowding).

(Finley noted that the algorithms had excellent teaching information. However, in a six-minute time frame, nurses had no time to read the information, or to read it to callers.)

**Remote Triage Process**

**Clerical Staff**: Patients contacting the call center encounter Health Care Associates (HCA) as the first point of contact. HCAs fielded the initial calls and consulted lists of urgent symptoms (Chest Pain, Difficulty breathing, etc.) that prompted HCAs to immediately transfer the call to the nurse.

**Clinical Decision-Making Process**

Although some nurses initially performed a symptom history initially, management did not require it. Nurses typically choose the algorithm that matches the patient’s first or key symptom. Nurses follow that algorithm completely to rule out most serious symptoms. They might then go on to a second or third algorithm.

(Finley observed that nurses were discouraged from using their own judgement and are required to follow electronic algorithms. However, nurses could use their own discretion to override algorithms and to upgrade the patient or symptom urgency.)

COMMENT: There is a risk associated with failure to perform an adequate initial assessment –cognitive errors of jumping to conclusions and many others.

**ED Physicians** served as a second-level triage – approving ED on-site evaluations – when nurses requested.

**Electronic Medical Record and Documentation:** Audiotaped conversations were saved as an electronic paper trail for later discussion at staff meetings. The EHR had provisions to track repeat calls regarding similar symptoms. It also verified, corrected, and updated EHR patient data (Medications, Allergies, Previous Medical Diagnoses, recent lab results, surgeries, procedures, Pregnancy or Breast-feeding status, History of Cancer, or Immunosuppression).

**Quality Assurance and Standards** Quality assurance consisted of a review of calls, typically prompted by patient complaints, or the need to update Algorithms. Nurses developed and revised the standards and algorithms. Monthly Evaluations involved “safety checks” performed for any bad outcomes regarding 3 things:

1. Overly lengthy calls (Timing Improvement)
2. Algorithm(s) Used.
3. If a nurse failed to make a Call Back Statement (regarding worsening symptoms or new symptoms) to the patient

**Policy Changes**

**Documentation –** During her time as an employee, Finley stated that there was no opportunity to perform a preliminary assessment of the symptom(s) or a place to document that information. A frequent outcome of this policy was that physicians who later evaluated the patient on-site had little or no idea why the patient was given an appointment – a major disconnect and lack of continuity.

A new RHS policy provides for both time to document and a free text section for Nursing Notes (using an acronym to standardize data collection) to note patients’ reasons for contacting the call center – a short patient statement about the symptom history.

**Patient Follow Up Call** -As part of the process, nurses now initiate follow up calls to patients to inquire about their progress after the call, thus enhancing patient satisfaction.

**Nurse Televisit or Message to –**During the COVID pandemic, call centers experienced high call volume. A new policy emerged as a result. Patients were offered the option to request a virtual nurse televisit or televisit call-back. (Finley notes that because some calls are as urgent as those to an ED, potential delays in care could inadvertently occur while patients unknowingly wait for the televisit or call about symptoms they do not recognize as serious.

**Ms. Finley’s Perspective**

During her time at the clinical call center, Finley experienced disappointment and job dissatisfaction. She decided to leave after five years and gave several reasons for this decision.

She felt that clinical decision making was reduced to a menial task. Finley wanted more clinical training and more professional autonomy. She stated, “the more clinical training the better.”

She felt a lack of management support for staff problem solving or decision making and to make patient care decisions. She noted that managers often micro-managed staff, leaving her feeling humiliated. She later felt trapped in a role that required her to perform a task controlled by management who had no vision and offered little or no staff support.

Finley “was not sure she was practicing nursing at times”, adding that management “had little bedside experience and overemphasized the need for speed”. She described typical nurses as both clinicians and “people persons” but sensed that the system discouraged her from showing human warmth (i.e., introducing herself, etc.) or from getting a preliminary clinical sense of why the patient called, or the patient’s level of anxiety as evidenced by their words, tone of voice and cadence of speech.

Finley maintained that “Good care makes for good patient outcomes” and that (in the U.S.) “nurses are the most trusted clinicians and regarded as comforting, understanding and supportive. She added that, “at the bedside, increased physical contact equates to increased intimacy, enhanced communication, and trust-building”. She stressed that “the more management overemphasizes the bottom line (cost savings related to rapid patient-clinician interactions) the more laborious it is for patient to trust the nurse”.

On the plus side, Finley believes the RHS electronic algorithms are good and that asking mandatory questions helps reduce risk. She stated that once a patient had been admitted, evaluated, and diagnosed, the RHS care was excellent. As a patient of the RHS system, she notes that when finally admitted, care was very timely. Patients received diagnostic tests, referrals to specialists, treatments, and appointments quickly.

Finley states that RHS clinical call centers are “regarded by many in the healthcare industry as the gold standard in terms of organization sophistication. She believes that the RHS EMR is “state of the art”, adding that industry leaders and patients alike regard it as extremely technologically advanced.

Finley felt that cost containment versus patient care is not necessarily an either/or but a both/and situation. She stated that many of the changes she envisioned were later instituted after she left the job. She adds that despite these advances, some system components and policies need further development and improvement.

**JOB SATISFACTION**

Job satisfaction is defined as a synthesis of experienced meaningfulness, experienced responsibility, and knowledge of results. Coincidentally, Finley’s job satisfaction on skill variety, autonomy and feedback was quite low. Finley’s job satisfaction self-reported the degree to which the job:

- requires completion of a whole and identifiable task or process - **Task identity** – 9/10
- has substantial impact on the lives or work of other people - **Task significance** – 9/10
- requires a clinician to perform a variety of different activities using different skills and talents - **Skill variety** – 5/10
- results in the individual obtaining direct and clear information about the effectiveness. **Feedback** - 5/10
- allows clinicians substantial freedom, independence, and discretion in carrying out the task. **Autonomy** 3/10

COMMENT: Finley’s low satisfaction scores beg the question of whether a more clearly defined and developed system -- structure, process, outcomes including components (Donabedian), might enhance job understanding, satisfaction and patient outcomes.

For example, per a U.S. patient privacy policy - Health Insurance Portability and Accountability Act (HIPAA) - call center nurses are not allowed access to finally diagnoses - to learn the outcome of their decisions. They essentially work in a vacuum. Might not feedback about the final diagnosis help nurses learn from their mistakes, or successes, thereby improving performance and job satisfaction?
______________
